# Acid sphingomyelinase-dependent autophagic degradation of GPX4 is critical for the execution of ferroptosis

**DOI:** 10.1038/s41419-020-03297-w

**Published:** 2021-01-07

**Authors:** Faisal Thayyullathil, Anees Rahman Cheratta, Ameer Alakkal, Karthikeyan Subburayan, Siraj Pallichankandy, Yusuf A. Hannun, Sehamuddin Galadari

**Affiliations:** 1 Cell Death Signaling Laboratory, Division of Science (Biology), Experimental Research Building, New York University Abu Dhabi, P. O. Box. 129188, Abu Dhabi, UAE; 2 Departments of Medicine and Biochemistry, Stony Brook University, Stony Brook, New York, 11794 USA

**Keywords:** Lipid peroxides, Cancer models, Macroautophagy, Lipid signalling

## Abstract

Ferroptosis is a type of regulated cell death characterized by ROS accumulation and devastating lipid peroxidation (LPO). The role of acid sphingomyelinase (ASM), a key enzyme in sphingolipid metabolism, in the induction of apoptosis has been studied; however, to date its role in ferroptosis is unclear. In this study, we report that ASM plays a hitherto unanticipated role in promoting ferroptosis. Mechanistically, Erastin (Era) treatment results in the activation of ASM and generation of ceramide, which are required for the Era-induced reactive oxygen species (ROS) generation and LPO. Inhibition of nicotinamide adenine dinucleotide phosphate oxidase (NADPH oxidase) or removal of intracellular ROS, significantly reduced Era-induced ASM activation, suggesting that NADPH oxidase-derived ROS regulated ASM-initiated redox signaling in a positive feedback manner. Moreover, ASM-mediated activation of autophagy plays a critical role in ferroptosis inducers (FINs)-induced glutathione peroxidase 4 (GPX4) degradation and ferroptosis activation. Genetic or pharmacological inhibition of ASM diminishes Era-induced features of autophagy, GPX4 degradation, LPO, and subsequent ferroptosis. Importantly, genetic activation of ASM increases ferroptosis in cancer cells induced by various FINs. Collectively, these findings reveal that ASM plays a novel role in ferroptosis that could be exploited to improve pathological conditions that link to ferroptosis.

## Facts

- Ceramide is generated during the process of ferroptosis.
- ASM-mediated redox amplification is critical for ferroptosis.
- ASM regulate autophagic degradation of GPX4 and ferroptosis.

## Introduction

Active cell death is a fundamental biological event, and it plays a major role in various pathophysiological processes^1,2^. Studies over the past several decades have explored and elucidated several of the key cell death (basically apoptotic and non-apoptotic) pathways. Amongst them, ferroptosis has recently been identified as a non-apoptotic cell death pathway driven by iron-mediated production of reactive oxygen species (ROS) and subsequent lipid peroxidation (LPO)^3^. The sensitivity to ferroptosis is tightly linked to numerous biological processes, including (i) amino acid, iron, and polyunsaturated fatty acid metabolism and (ii) biosynthesis of glutathione (GSH), phospholipids, nicotinamide adenine dinucleotide phosphate (NADPH), and coenzyme Q10^4^. Small-molecule ferroptosis inducers (FINs) such as erastin (Era), sulfasalazine (SLS), 1S,3R-Ras Selective Lethal 3 (RSL3), and N2, N7-dicyclohexyl-9-(hydroxyimino)-9H-fluorene-2,7-disulfonamide (FIN56) have been established as pharmacological activators of ferroptosis^5^. Ferroptosis has been implicated in the pathological cell death associated with neurodegenerative diseases (i.e., Alzheimer’s, Huntington’s, and Parkinson’s diseases), stroke, intracerebral hemorrhage, traumatic brain injury, ischemia-reperfusion injury, myocardial infarction, and kidney degeneration^4–6^. Ferroptosis may also have tumor-suppressor function that could be exploited for anticancer therapy^4,5^. Thus, an improved understanding of key players in ferroptosis will provide new opportunities for diagnosis and therapeutic intervention in alleviating human diseases including cancer, neurodegeneration, and ischemic diseases^4,5^.

Glutathione peroxidase 4 (GPX4) is a central defense enzyme against LPO and ferroptosis^7,8^. A deficiency in GSH (a tripeptide antioxidant that contains a cysteine in the center) causes a decrease in GPX4 activity, which leads to elevated LPO and ferroptosis^9^. Thus, cysteine availability, GSH biosynthesis, and proper functioning of GPX4 are the key cellular processes in inhibiting ferroptosis. On the other hand, conditions that negatively regulate GPX4 level or activity can sensitize or even trigger ferroptosis^7,8,10,11^. FINs such as Era, SLS, RSL3, and FIN56 cause GPX4 degradation and LPO, leading to ferroptosis^12–15^. Moreover, overexpression of GPX4 confers resistance to FINs-induced ferroptosis, suggesting that GPX4 degradation plays an important role in the induction of ferroptosis^7,12^.

Autophagy is an evolutionarily conserved lysosomal degradative pathway that plays a complex role in various pathophysiological conditions^16–18^. During autophagy, damaged or obsolete proteins/subcellular organelles are sequestered within a double-membraned vesicle called autophagosomes. These autophagosomes subsequently fuse with lysosomes (forming autolysosomes), leading to degradation of the sequestered contents^19^. At the molecular level, autophagy is executed by a set of autophagy-related (ATG) proteins^16^. Depending on the context, autophagy promotes either cell survival or cell death^16–18^. Recent evidence suggests that autophagy also plays a crucial role in the execution of ferroptosis. However, the precise molecular mechanisms that link autophagy to ferroptosis remain poorly understood.

Ceramide is a central molecule in sphingolipid metabolism that facilitates a variety of tumor suppressive programs, such as apoptosis, autophagy, senescence, and necroptosis^20–22^. Ceramide can be generated through the de novo synthesis pathway, salvage of sphingosine, or hydrolysis of sphingomyelin^20^. The latter reaction involves the action of sphingomyelinases (SMases), a class of enzymes that differ in their cellular localization and pH optima for activity^20,23^. Acid sphingomyelinase (ASM) was the first SMase to be identified and characterized^24^. Extensive research on this enzyme has revealed its role in a variety of pathophysiological conditions^25^. ASM is activated by a diverse array of stresses such as heat shock, UV irradiation, oxidative stress, and anticancer drugs^21,23,26–28^. It was demonstrated that cells or mice deficient of ASM resist the induction of apoptosis by anticancer stimuli^29,30^. Conversely, overexpressing ASM alone increases the sensitivity of cancer cells to chemotherapy and radiotherapy^31,32^, indicating that ASM is a potential target for anticancer therapy. Significant research has focused on apoptosis inducing tumoricidal functions of ASM. Although, ASM has been shown to promote mitochondrial dysfunction in glutamate-induced ferroptosis of oligodendrocytes^33^, the underlying mechanisms by which ASM regulates ferroptosis is still unclear.

In this study, we report that ASM plays a crucial role in promoting ferroptosis. Particularly, we demonstrate that ASM-mediated redox amplification is required for the autophagic degradation of GPX4, leading to subsequent LPO and ferroptosis. Thus, ASM contributes to the core molecular machinery and signaling pathways involved in ferroptosis.

## Materials and methods

### Chemicals and antibodies

3-[4,5-dimethylthiazol-2-yl]-2,5-diphenyl tetrazolium bromide (MTT), 5,5′-dithiobis-(2-nitrobenzoic acid (DTNB), GSH, GSH assay kit, N-acetyl cysteine (NAC), superoxide dismutase (SOD), catalase (Cat), phthaldialdehyde (OPA), Era, RSL3, FIN56, desipramine hydrochloride (Des), GW4869, brefeldin A (BFA), ferrostatin-1 (Fer-1), deferoxamine (DFO), rapamycin, bafilomycin A1 (BafA1), hydroxychloroquine (HCQ), ammonium chloride (NH_4_Cl), 2′,7′-dichlorodihydrofluorescein diacetate (DCFH-DA), dithiothreitol (DTT), ethylene diamine tetra acetic acid (EDTA), phenyl methyl sulfonyl fluoride (PMSF), and dimethyl sulfoxide (DMSO) were purchased from Sigma Chemical Co. (St. Louis, MO, USA). Dulbecco’s modified essential medium (DMEM), Opti-MEM, McCoy’s 5A medium, phosphate-buffered saline (PBS), trypsin–EDTA, penicillin–streptomycin (10,000 U/mL), and fetal bovine serum (FBS) were purchased from Gibco BRL (Grand Island, NY, USA). Fumonisin B1 (FB1), myriocin (Myr), zoledronic acid monohydrate (ZA), C6-NBD-sphingomyelin, MG132, diphenyleneiodonium chloride (DPI), apocynin (Apo), and Mito TEMPO (MT) were purchased from Santa Cruz Biotechnology Inc. (Santa Cruz, CA, USA). Anti-SLC7A11 (#12691S), and anti-LC3B (D11) XP (#3868) antibodies were from Cell Signaling Technology (Beverly, MA, USA). Anti-LC3B (#L7543), anti-rabbit IgG (#A6154), and anti-mouse IgG (#A0412) were purchased from Sigma Chemical Co. (St. Louis, MO, USA). Anti-actin (#sc-1616; #sc-47778), anti-p62/SQSTM1 (#sc-28359), anti-Beclin-1 (#sc-11427), and donkey anti-goat IgG (#sc-2056) antibodies were from Santa Cruz Biotechnology Inc. (Santa Cruz, CA, USA). Anti-GPX4 (#125066) was purchased from Abcam, Cambridge, MA, USA. Anti-DDK (#TA50011-1) and MegaTran 1.0 transfection reagent (#TT200002) were from Origene Technologies, Inc. (Rockville, MD, USA). Peroxidase affinity pure goat anti-mouse HRP (#115-035-003) antibodies were from Jackson Immuno Research Europe Ltd. (Cambridge House, UK).

### Cell culture conditions and drug treatment

Human fibrosarcoma (HT-1080), human lung carcinoma (Calu-1) cells were purchased from European Collection of Authenticated Cell Cultures (ECACC) (Porton Down Salisbury, UK), and human cervical cancer (HeLa) cells were procured from ATCC (Rockville, MD, USA). HT-1080 and HeLa cells were cultured in DMEM. Calu-1 cells were cultured in McCoy’s 5A media. All cells were supplemented with 10% heat-inactivated FBS, 50 IU/mL penicillin, and 50 μg/mL streptomycin in an incubator containing humidified atmosphere of 95% air and 5% CO_2_ at 37 °C. Cells were checked quarterly for mycoplasma contamination using the first generation MycoAlert^TM^ mycoplasma detection kit (Lonza, #: LT07-118). Era (10 mM), SLS (500 mM), RSL3 (5 mM), and FIN56 (5 mM) stock solution in DMSO were prepared and stored in a dark-colored bottle, from which desired dilutions were made. Cells were grown to about 70–80% confluence and then treated with drugs at required concentrations and for a different time period.

### Cell viability assay

Cell viability assay was carried out as described previously^34^. Briefly, cells (10,000 cells/well) were plated in 96-well flat-bottom plates and were subjected to drug treatment. Subsequently, at desired time intervals, 25 μL of MTT (5 mg/mL) in PBS was added to each well. The plates were incubated for an additional 2 h at 37 °C. After the incubation, the formazan crystals were solubilized in 200 μL of DMSO and the absorbance at 570 nm was measured using EnSpire™ multimode plate reader (PerkinElmer, Waltham, USA).

### Live/Dead assay

The numbers of live and dead cells were assessed using a Live/Dead assay Kit (Thermo Scientific, CA, USA) according to the manufacturer’s protocol. In brief, Era exposed cells were incubated with 2 μM calcein and 4 μM ethidium homodimer-1 mixture in the dark. After 30 min incubation at 37 °C, images of live and dead cells were captured using IX73 inverted fluorescent microscope (Olympus, Tokyo, Japan).

### Protein lysate preparation and immunoblot analysis

Cells were washed twice with PBS and lysed in a RIPA lysis buffer (50 mM Tris–HCl pH 7.4, 1% NP-40, 40 mM NaF, 10 mM NaCl, 10 mM Na_3_VO_4_, 1 mM PMSF, 10 mM DTT, and EDTA-free protease inhibitor tablet). The cell lysates were centrifuged and total protein were determined by Bio-Rad protein assay. Lysates were mixed with 6× loading buffer and boiled at 100 °C for 3 min. Samples at 30–50 μg/lane were resolved by SDS–PAGE and the separated proteins were transferred on to nitrocellulose membrane by wet transfer method using Bio-Rad electro transfer apparatus. Following the transfer, blots were blocked with 5% non-fat milk in Tris-buffer saline containing 0.1% Tween-20. Blots were then incubated with primary antibodies followed by secondary antibody. Protein bands were visualized using Super Signal West Pico Chemiluminescence reagent (Thermo Scientific, CA, USA). Densitometry analyses were performed by normalizing target protein bands to their respective loading control using Image Studio Lite software (LI-COR Biosciences).

### Intracellular ROS measurement

Generation of intracellular ROS was measured by the oxidation-sensitive fluorescent probe DCFH-DA. After the treatment, cells were washed twice with PBS and then supplied with 25 μM of DCFH-DA for 30 min. The cells were then washed 3–4 times with PBS to remove the extracellular dye, harvested in phenol red free medium, and was assayed for DCF fluorescence with an excitation wavelength of 485 nm and an emission wavelength of 535 nm using EnSpire™ multimode plate reader (PerkinElmer, Waltham, USA).

### LPO assay

Image-iT LPO Kit (Thermo Scientific, CA, USA) was used to measure the LPO. Briefly, after the treatment, cells were washed with PBS, trypsinized, and centrifuged. The pellet was stained with 2 µM C11-BODIPY 581/591 for 30 min at 37 °C. Oxidation of the polyunsaturated butadienyl portion of the dye causes a fluorescence emission peak shift from ~590 to ~510 nm, detected using flow cytometric analysis (BD FACSAriaIII; Becton Dickinson, Heidelberg, Germany). The fluorescence signal was then quantitated using FlowJo V.10.1 software.

### Determination of total GSH

GSH levels were determined as detailed in the manufacturer’s protocol. Briefly, after the treatment, the cells were lysed in 150 μL of lysis buffer (5% sulfosalicylic acid). Lysates were centrifuged and 50 μL of supernatant was mixed with 150 μL of assay buffer (potassium phosphate buffer, pH 7.0, containing 5 mM EDTA, 1.5 mg/mL DTNB, and 6 U/mL GSH reductase). To this mixture, 50 μL (0.16 mg/mL) of NADPH in potassium phosphate buffer was added and absorbance was measured at 412 nm using EnSpire™ multimode plate reader (PerkinElmer, Waltham, USA).

### ASM activity assay

Assays for ASM activity was performed as described previously^35^. Briefly, after the treatment, the cells were lysed in 150 µL of ASM assay lysis buffer (250 mM sodium-acetate, 0.2% Triton X-100, pH 5.0), 5 μg of protein were mixed with ASM reaction buffer (100 mM acetate buffer pH 5, 10 μM C6-NBD-sphingomyelin, and 0.1% Triton X-100), and final volume of 100 µL was made using lysis buffer. The mixture was then incubated at 37 °C for 90 min. The reaction was stopped by the addition of 90 μL H_2_O and 200 μL chloroform and methanol (2:1; v/v), mixed well, and centrifuged. Lower phase was collected, solvent was evaporated, aliquots were applied on to TLC plates, and developed with a solvent consisting of chloroform:methanol:12 mM MgCl_2_ (65:25:4 v/v/v). The spot corresponding to C6-NBD-Ceramide were scraped, incubated with ethanol at 37 °C for 5 min to extract the compounds from silica, and fluorescence was measured at 485/535 nm excitation/emission wave length using EnSpire™ multimode plate reader (PerkinElmer, Waltham, USA).

### Ceramide measurement

Ceramide was measured as described previously^36^. After the treatment, cells were washed with PBS and lysed using ceramide assay lysis buffer (50 mM Tris pH-7.4 containing 0.2% IGEPAL CA 630) by freeze and thaw method. The lysate was then heated at 70 °C for 5 min and centrifuged at 12,000 rpm for 10 min at 4 °C. The reaction was started by mixing 10 µL of supernatant with 10 ng of recombinant human neutral ceramidase enzyme. After incubation for 1 h at 37 °C, the reaction was stopped by adding 55 μL of stopping buffer (1:9, 0.07 M potassium hydrogen phosphate buffer: methanol), and the released sphingosine was derivatized with OPA. After incubation for 30 min at room temperature in the dark, an aliquot of 25 μL was injected, and HPLC for the ceramide analysis was conducted using Waters 1525 binary pump system, Waters XTerra RP18 (5 μm, 3 × 250 mm) column, and Waters 2475 fluorescence detector at an excitation wavelength of 340 nm and an emission wavelength of 455 nm. The mobile phase (20% methanol, 80% 1:9 stopping buffer) was used at a flow rate of 0.5 mL/min.

### Analyses of endogenous sphingolipids

Sphingolipid analysis was custom-performed by Creative Proteomics (Shirley, NY). Briefly, after the drug treatment, pelleted cells were combined with 1440 μL of ice-cold 75% methanol, 270 μL of chloroform, and 20 μL of a mixture of C17 and C12 sphingolipid internal standards, each at a concentration of 25 nmol/mL (Avanti Polar Lipids Cer/Sph Mixture II). Samples were then homogenized with 0.5 mm zirconium oxide beads in a bullet blender sample homogenizer. After the addition of methanolic potassium hydroxide (0.1 N), samples were vortexed for 30 min and then heated for 2 h at 37 °C. The sample were then centrifuged and supernatants were collected to a new test tube. Cellular pellets were re-extracted and pooled with the first extract. The pooled supernatants were dried under vacuum and the pelleted salts and debris were subsequently re-extracted twice with chloroform:methanol (1:2 v/v). The pooled final extracts were dried again under vacuum and resuspended in 200 μL of methanol. Sphingolipids were analyzed by high resolution/accurate mass (HRAM)–LC–MS using a Shimadzu Prominence HPLC coupled to a Thermo Scientific LTQ-Orbitrap Velos mass spectrometer. The LC column was a Phenomenex HILIC 2.0 mm × 100 mm column with 3.0 micron particles and 100 Angstrom pore size, fitted with a guard cartridge of matching chemistry. Solvent A was water containing 50 mM ammonium formate and Solvent B was acetonitrile (100%). The flow rate was 200 μL/min and the column oven was held at 50 °C. 5 μL of each sample was injected and separated by gradient elution. Column eluent was introduced to a Thermo LTQ-Orbitrap Velos mass spectrometer via a heated electrospray ionization source. The mass spectrometer was operated in positive ion mode at 60,000 resolutions with data-dependent MS/MS at 30,000 resolution using higher energy collisional dissociation. The electrospray ionization source was maintained at a spray voltage of 4.5 kV with sheath gas at 30 (arbitrary units) and auxiliary gas at 10 (arbitrary units). The inlet of the mass spectrometer was held at 300 °C and the S-lense was set to 50%. The heated ESI source was maintained at 300 °C. Chromatographic peak alignment, peak finding, peak identification, isotopic corrections, and peak area calculations were performed with MAVEN software.

### Plasmids and transient transfection

pCMV6-Myc-DDK-tagged ASM (#RC219758) and pCMV Entry-Myc-DDK empty vector (#PS100001) were purchased from OriGene Technologies, Inc. (Rockville, MD, USA). Dual fluorescent mRFP-EGFP-LC3 (ptfLC3) (Addgene plasmid #21074) was kind gift from Tamotsu Yoshimori. DNA transfection to cells was performed by using MegaTran 1.0 transfection reagent as described in the manufacture’s protocol.

### Small interfering RNA transfection

siControl (#sc-37007) and siAtg5 (#sc-41445) were purchased from Santa Cruz Biotechnology Inc. (Santa Cruz Biotechnology, CA, USA). For the transfection, cells were seeded into six-well plate at a density of 0.2 × 10^6^ cells per well and allowed to reach ~50% confluence on the day of transfection. Cells were transfected with 30 nM siRNA using HiPerFect transfection reagent (Qiagen, Valencia, CA, USA) as described in the manufacturer’s protocol.

### shRNA-mediated knock-down of ASM

shRNAs for ASM constructs in lentiviral GFP vector (#TL309232) were purchased from OriGene Technologies (Rockville, MD, USA):

ASM-shRNA1: ACCGAATTGTAGCCAGGTATGAGAACACC

ASM-shRNA2: GGAACATCTCTTTGCCTACTGTGCCGAAG

Cont-shRNA: GCACTACCAGAGCTAACTCAGATAGTACT

Lentiviruses carrying the shRNAs were produced in 293T cells using a Lenti-Pac expression packing kit (Genecopoeia, Rockville, MD, USA) according to manufacturer’s instruction. HT-1080 cells were infected by these viruses and were selected with 0.2 μg/mL puromycin. Scrambled control shRNA was used as a control.

### Determination of autophagic flux

Tandem reporter construct mRFP-GFP-LC3 (ptfLC3) was used for monitoring autophagic flux based on different pH stability of GFP and mRFP fluorescent proteins. GFP is quenched in acidic environments, while mRFP is relatively stable at the acidic pH found in lysosomes. Therefore, the formation of autophagosomes leads to an increase of yellow puncta (GFP+/RFP+), with these puncta turning red (GFP−/RFP+) upon fusion of an autophagosome with a lysosome. An increase in autophagy leads to more yellow and red puncta, while a block at fusion of autophagosomes with lysosomes leads to an increase in yellow puncta but a decrease in red puncta. HT-1080 cells transfected with ptfLC3 were treated with Des in the presence of absence of Era for 10 h. Following the treatment, fluorescence images were taken under Olympus DP71 fluorescent microscope. Cells treated with 1 µM rapamycin for 48 h served as positive control while cells treated with BafA1 served as negative control for autophagy. Autophagy was determined by quantification of the number of cells with LC3-positive organelles, counting at least 20 cells in triplicate per condition.

### Statistical analysis

Statistical analysis was performed using Graph Pad Prism 8.0 software. Data shown are mean ± standard deviation (*n* = 3). Statistical significance was analyzed by one-way ANOVA using the Bonferroni post-hoc test. Asterisk (*) represents *p*-value < 0.05, double-asterisk (**) represents *p*-value < 0.01, and triple-asterisk (***) represents *p*-value < 0.001. Differences were considered significant only when *p* < 0.05.

## Results

### Ceramide accumulates during the process of ferroptosis

**Fig. 1:**
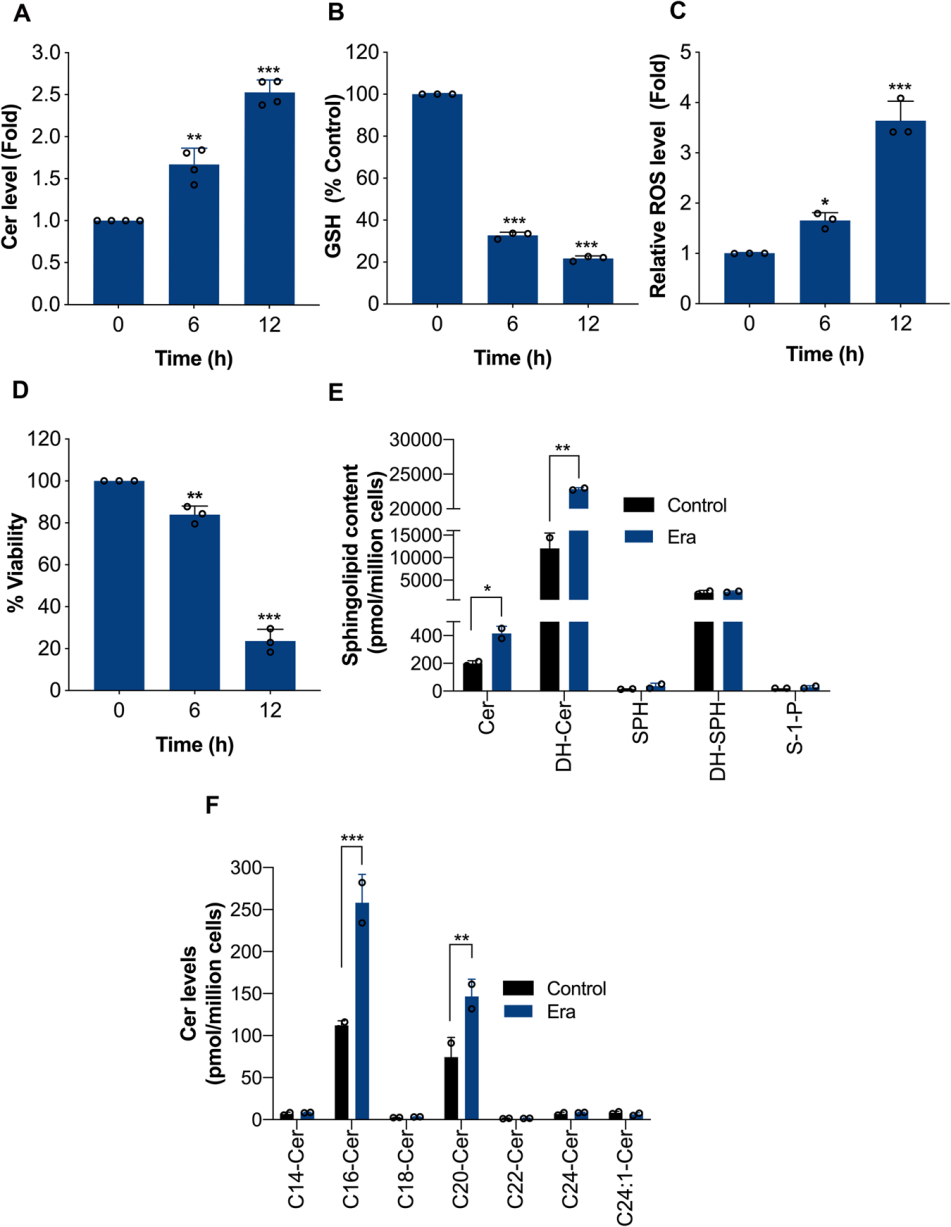
Ceramide is generated during the process of ferroptosis. HT-1080 cells were treated with Era (10 μM) for the indicated time period. Following the treatment, measured the levels of **A** ceramide using HPLC. Data shown are mean ± SD (*n* = 4), **B** GSH, **C** ROS using DCFH-DA fluorescent dye, and **D** cell viability by MTT assay. Data shown are mean ± SD (*n* = 3). Significant differences, *p < 0.05, **p < 0.01, and ***p < 0.001 versus control. HT-1080 cells were treated with Era (10 μM) for 12 h. Lipids were extracted and levels of **E** indicated sphingolipids and **F** various ceramide species were determined as described in the “Materials and methods” section. Lipids were expressed as pmol/million cells. Data shown are mean ± SD (*n* = 2). Significant differences, *p < 0.05, **p < 0.01, and ***p < 0.001 versus respective control.

Ceramide is a central molecule in the sphingolipid metabolic pathway that facilitates a variety of tumor-suppressive programs such as apoptosis, autophagy, and necroptosis^21^. Ceramide can be activated in response to a diverse array of stressors including genotoxic damage, inflammatory mediators, heat shock, oxidative stress, and anticancer drugs^21^. Significant research has focused on apoptosis, autophagy, and necroptosis inducing tumoricidal functions of ceramide. Recently, it has been reported that ceramide (C16-ceramide) is accumulated during the process of piperazine Era-induced ferroptosis^37^. However, the involvement of ceramide in regulating ferroptosis is poorly defined. In order to investigate the role of ceramide in ferroptosis, we used well-established ferroptosis model cell lines, human fibrosarcoma (HT-1080), human lung cancer (Calu-1), and human cervical cancer (HeLa) cells, in which classical FINs such as Era, SLS, RSL3, and FIN56 have been reported to induce ferroptosis^37,38^. First, we treated HT-1080 cells with Era (10 μM) for different time period and the total ceramide levels were determined by using HPLC. A significant accumulation of total ceramide was detected following the Era exposure (Fig. 1A). Importantly, ceramide accumulation is an early event during ferroptosis, and it occurs with similar time-course to GSH depletion (Fig. 1B), ROS generation (Fig. 1C), and loss of viability (Fig. 1D). Additionally, lipid extraction and mass spectrometry analysis were carried out on these Era (10 μM)-treated cells. As shown in Fig. 1E, ceramide (Cer) and dihydroceramide (DH-Cer) levels were elevated in Era-treated HT-1080 cells. On the other hand, levels of other sphingolipids such as sphingosine (SPH), dihydrosphingosine (DH-SPH), and sphingosine-1-phosphate (S-1-P) were unchanged. Analyses of the various ceramide species revealed that Era induced a robust elevation in C16-ceramide and C20-ceramide, and no change in the levels of C14-, C18-, C22-, C24-, and C24:1-ceramide (Fig. 1F). These results demonstrate significant effects of Era on ceramide metabolism. Similar to HT-1080 cells, Era (2 μM) also induced ceramide accumulation in Calu-1 cells (Supplementary Fig. 1). Next, we investigated whether other types of FINs such as RSL3 and FIN56 can induce ceramide accumulation. As shown in Supplementary Fig. 1, treatment of HT-1080 and Calu-1 cells with RSL3 (2 μM) and FIN56 (4 μM) induced significant ceramide accumulation, suggesting that ceramide is accumulated during the process of ferroptosis irrespective of the inducer.

### ASM-dependent ceramide generation is required for the ferroptosis

**Fig. 2:**
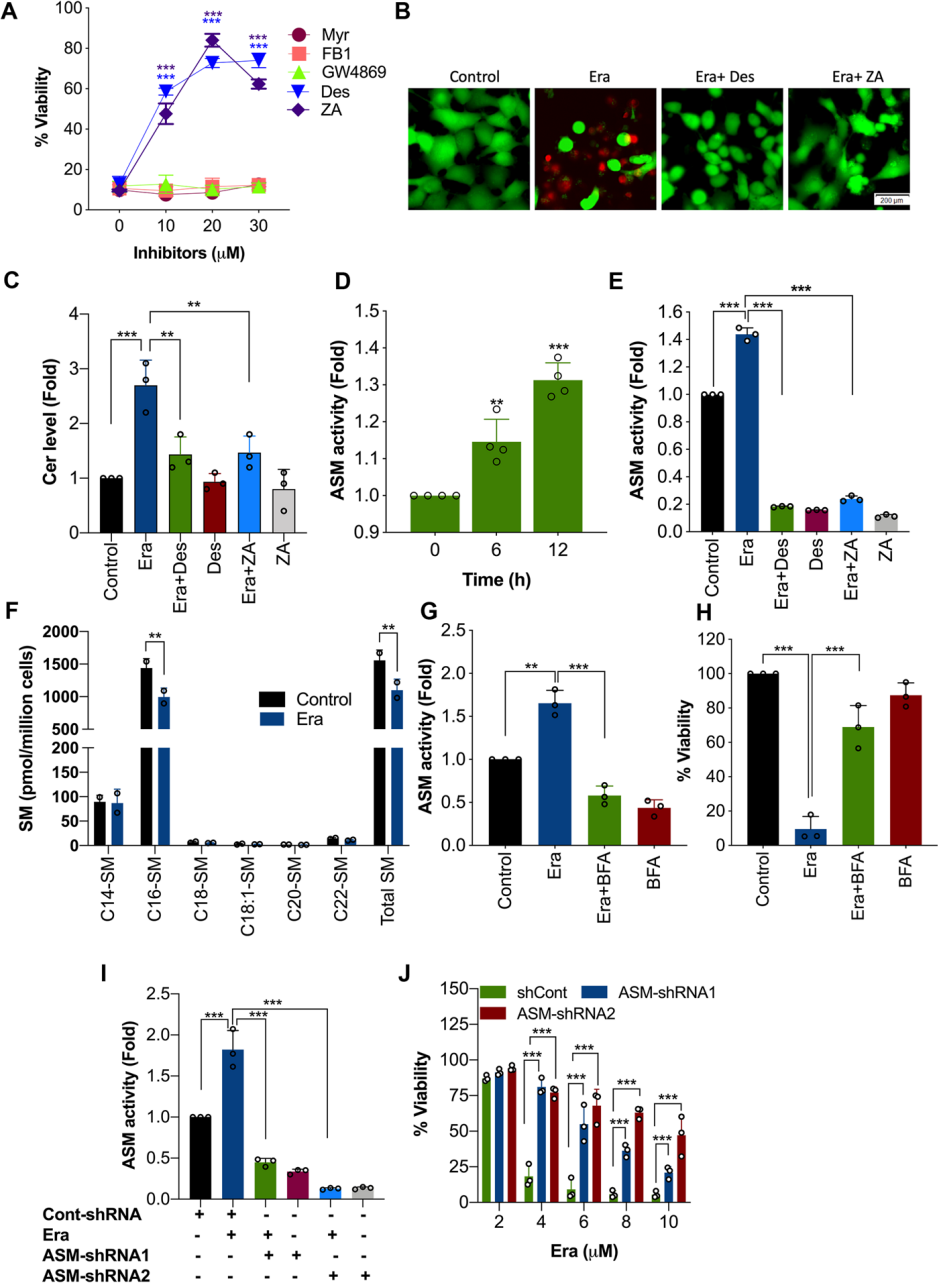
ASM is involved in Era-induced ceramide accumulation and ferroptosis. **A** HT-1080 cells were treated with increasing concentration of inhibitors in the presence or absence of Era (10 μM) for 12 h. Cell viability was measured by MTT assay. Data shown are mean ± SD (*n* = 3). Significant differences, ***p < 0.001 versus respective control. HT-1080 cells were treated with Era (10 μM) in the presence or absence of Des (10 μM) and ZA (20 μM) for 12 h. Following the treatment, **B** live–dead assay was performed and **C** ceramide analysis was performed using HPLC. Data shown are mean ± SD (*n* = 3). Significant differences, **p < 0.01, and ***p < 0.001. **D** HT-1080 cells were treated with Era (10 μM) for the indicated time period and ASM activity assay was carried out. Data shown are mean ± SD (*n* = 4). Significant differences, **p < 0.01, and ***p < 0.001 versus res*p*ective control. **E** HT-1080 cells were treated with Era (10 μM) in the pr**e**sence or absence of Des (10 μM) and ZA (20 μM). ASM activity was assayed. Data shown are mean ± SD (*n* = 3). Significant differences, ***p < 0.001. **F** HT-1080 cells were treated with Era (10 μM) for 12 h. Lipids were extracted from the cells and the levels of various sphingomyelin species (expressed as pmol/million cells) were determined. Data shown are mean ± SD (*n* = 2). Significant differences, **p < 0.01 versus respective control. HT-1080 cells were treated with Era (10 μM) in the presence or absence of BFA (100 nM) for 12 h. Following the treatment, cells were analyzed for **G** ASM activity and **H** cell viability. Data shown are mean ± SD (*n* = 3). Significant differences, **p < 0.01 and ***p < 0.001. **I** ASM-shRNA-transfected HT-1080 cells were treated with Era (10 μM) for 12 h and ASM activity assay was carried out. **J** ASM-shRNA-transfected HT-1080 cells were treated with the indicated concentrations of Era for 12 h. Following the treatment cell viability by MTT assay was performed. Data shown are mean ± SD (*n* = 3). Significant difference ***p < 0.001.

Generation of ceramide mainly proceeds from de novo biosynthesis or hydrolysis of sphingomyelin (SM)^21^. In order to distinguish whether any of these ceramide biosynthetic pathways are involved in the Era-induced ferroptosis, we used inhibitors targeting these pathways prior to Era treatment. The de novo biosynthesis inhibitors FB1 and Myr and the neutral sphingomyelinase inhibitor (GW4869) had no effect on Era (10 μM)-induced loss of viability (Fig. 2A). Interestingly, ASM inhibitors ZA and Des significantly blocked the Era-induced cell death (Fig. 2A, B) and ceramide generation (Fig. 2C). Likewise, treatment of HT-1080 cells with Era (10 μM)-induced significant activation of ASM (Fig. 2D) and this effect was inhibited by Des and ZA (Fig. 2E). Further to this, a decrease in C16-sphingomyelin was observed (Fig. 2F). However, C18-, C18:1-, C20-, and C22- sphingomyelin levels were not altered (Fig. 2F). Taken together, these results indicate that Era induces ASM-mediated conversion of sphingomyelin to ceramide, which is important for its ferroptotic-inducing property.

After its synthesis in the endoplasmic reticulum (ER), ASM activation requires its transportation to Golgi and then to lysosomes via classical BFA-sensitive secretory pathway. Interestingly, disrupting this transport with BFA (100 nM) significantly abolished Era-induced ASM activity (Fig. 2G) and cell death (Fig. 2H). It should be noted that BFA alone caused reduction in ASM activity, as it is likely to inhibit both basal and Era-induced ASM activation (Fig. 2G). To prove conclusively that ASM is, indeed, required for Era-induced ferroptosis, an shRNA approach was utilized. The results showed that shRNAs targeting different regions of human ASM (ASM-shRNA1 and ASM-shRNA2) significantly decreased the Era-induced ASM activation (Fig. 2I) and ferroptosis (Fig. 2J).

Next, we investigated whether ASM inhibition affects the ferroptosis-inducing activity of Era in other cell types. ASM inhibitors prevented Era-induced cell death in Calu-1 (Supplementary Fig. 2A, B) and HeLa (Supplementary Fig. 2B) cells, indicating that the role of ASM in ferroptosis is not specific for HT-1080 cells. Simultaneously, inhibition of ASM activity also prevented ferroptosis by other FINs such as SLS and RSL3 (Supplementary Fig. 2C, D), indicating that ASM, indeed, plays a crucial role in ferroptosis in diverse settings.

### ASM activation is down-stream to Era-induced inhibition of system xc^−^ and GSH depletion

**Fig. 3:**
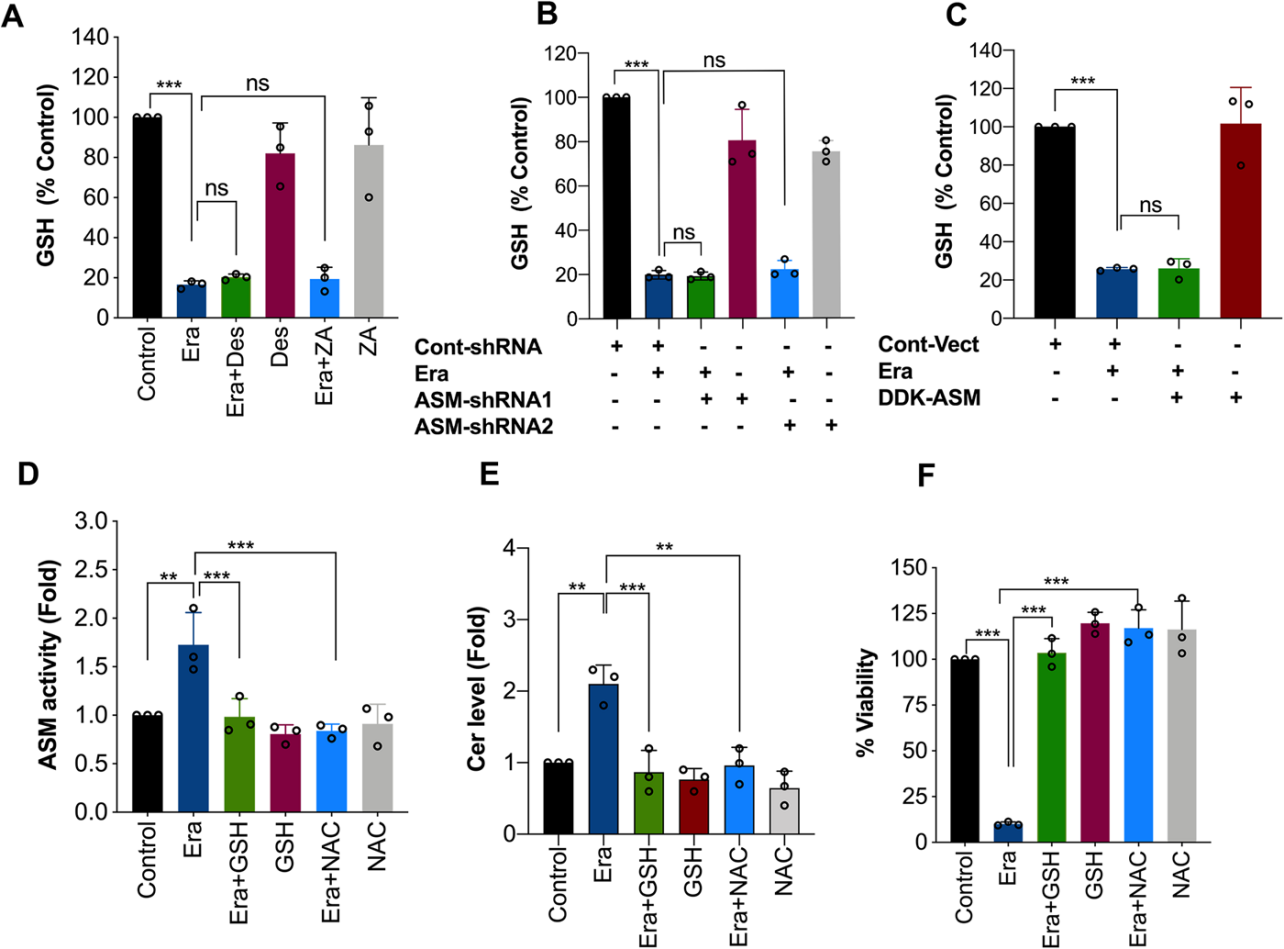
ASM activation is downstream to the Era-induced GSH depletion. **A** HT-1080 cells were treated with Era (10 μM) for 12 h in the presence or absence of Des (10 μM) and ZA (20 μM), **B** ASM-shRNA-transfected HT-1080 cells were treated with Era (10 μM), and **C** pCMV6-DDK-ASM-transfected HT-1080 cells were treated with Era (5 μM). Following the treatment, GSH assay was performed. Data shown are mean ± SD (*n* = 3). Significant differences, ***p < 0.001 and ns = non-significant. HT-1080 cells were treated with Era (10 μM) for 12 h in the presence or absence of GSH (5 mM) and NAC (5 mM). Following the treatment, **D** ASM activity assay, **E** ceramid**e** assay, and **F** MTT cell viability assay were performed. Data shown are mean ± SD (*n* = 3). Significant differences, **p < 0.01 and ***p < 0.001.

The cystine/glutamate antiporter (system xc^−^)-mediated cystine uptake is crucial for GSH synthesis, which is one of the major mechanisms that blocks LPO and ferroptosis^3,5^. System xc^−^ inhibition and corresponding GSH depletion is one of the decisive signaling events in the facilitation of Era-induced ferroptosis. As observed by others, Era (10 μM) exposure significantly attenuated GSH content (Figs. 1B and 3A). Importantly, inhibition of ASM activity (pharmacological or genetic) or activation of ASM activity by expressing DDK-ASM did not have any effect on Era-induced GSH depletion (Fig. 3A–C), indicating that ASM activation occurs downstream to the system xc^−^ inhibition and GSH depletion. To confirm this, we used GSH and its precursor NAC and examined whether they can inhibit Era-triggered ASM activation. As expected, Era-induced ASM activation (Fig. 3D), ceramide generation (Fig. 3E), and loss of viability (Fig. 3F) were completely prevented by both GSH and NAC. Altogether, these results suggest that Era-induces ASM activation down-stream to the inhibition of system xc^−^ and GSH depletion.

### ASM amplifies Era-induced ROS production

**Fig. 4:**
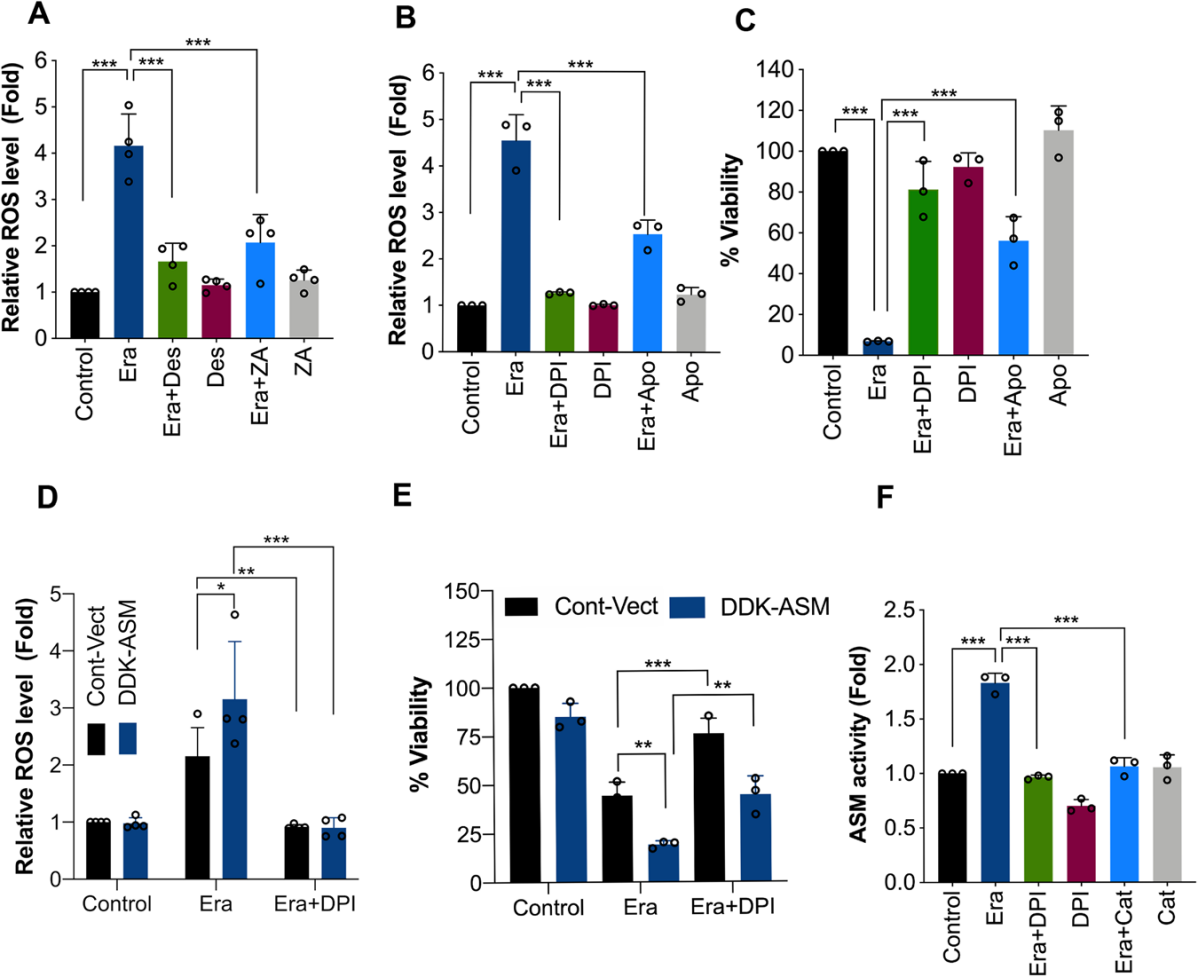
ASM-mediated ROS generation amplifies Era-induced ASM activation in a positive feedback manner. **A** HT-1080 cells were treated with Era (10 μM) for 12 h in the presence or absence of Des (10 μM) and ZA (20 μM). Following the treatment, ROS assay by using DCFH-DA was performed. Data shown are mean ± SD (*n* = 4). Significant differences, ***p < 0.001. HT-1080 cells were treated with Era (10 μM) for 12 h in the presence or absence of DPI (0.5 μM) and Apo (50 μM). Following the treatment, **B** ROS assay by using DCFH-DA, and **C** cell viability by MTT assay were performed. Data shown are mean ± SD (*n* = 3). Significant differences, ***p < 0.001. HT-1080 cells were stably transfected with pCMV6-DDK-ASM followed by treatment with Era (5 μM) in the presence or absence of DPI. Following the treatment, **D** ROS assay by using DCFH-DA was performed. Data shown are mean ± SD (*n* = 4). Significant differences, *p < 0.05, **p < 0.01, and ***p < 0.001, and **E** cell viability by MTT assay were performed. Data shown are mean ± SD (*n* = 3). Significant differences, **p < 0.01 and ***p < 0.001. **F** HT-1080 cells were treated with Era (10 μM) for 12 h in the presence or absence of DPI (0.5 μM) and Cat (2000 Units/mL). Following the treatment, ASM activity assay was performed. Data shown are mean ± SD (*n* = 3). Significant difference, ***p < 0.001.

NADPH oxidase-derived superoxide is a primary source of ROS generation during Era-induced ferroptosis^3^. Previously, ASM-mediated ceramide has been shown to induce ROS production in endothelial cells, macrophages, and hepatocytes^39–41^. Thus, we tested whether Era-induced ASM is crucial for Era-triggered ROS production and ferroptosis. As shown in Figs. 1C and 4A, Era-induced ROS burst in HT-1080 cells, whereas ROS release was significantly inhibited in cells pretreated with ASM inhibitors (Fig. 4A). Consistently, NADPH oxidase inhibitors, such as DPI (0.5 μM) and Apo (50 μM) significantly attenuated Era-induced ROS production and cell death (Fig. 4B, C). Similarly, mitochondria-specific superoxide scavenger MT (10 μM) and H_2_O_2_-specific scavenger Cat (2000 Units/mL) prevented Era-induced ROS production and cell death (Supplementary Fig. 3A, B). Therefore, NADPH oxidase-derived superoxide and its conversion product H_2_O_2_ are importantly involved in Era-induced ferroptosis. Moreover, overexpression of ASM amplified the Era-induced ROS production and ferroptosis, which was significantly diminished by DPI (Fig. 4D, E), suggesting that ASM is essential for Era-induced ROS production, an event critical for the induction of ferroptosis.

The data presented above indicate that ASM controls ROS generation upon Era-induced ferroptosis. However, redox regulation of ASM has also been well-documented in previous studies^40–42^. Therefore, we checked if there is a positive feedback regulation between ASM activation and ROS generation during Era-induced ferroptosis. As shown in Fig. 4F, Era-induced ASM activation was significantly inhibited by treatment with the NADPH oxidase inhibitor (DPI) and H_2_O_2_ scavenger (Cat). Together, these results indicate that ASM and ROS molecules are regulating each other in a positive feedback manner during Era-induced ferroptosis.

### ASM is critical for the Era-induced autophagy

**Fig. 5:**
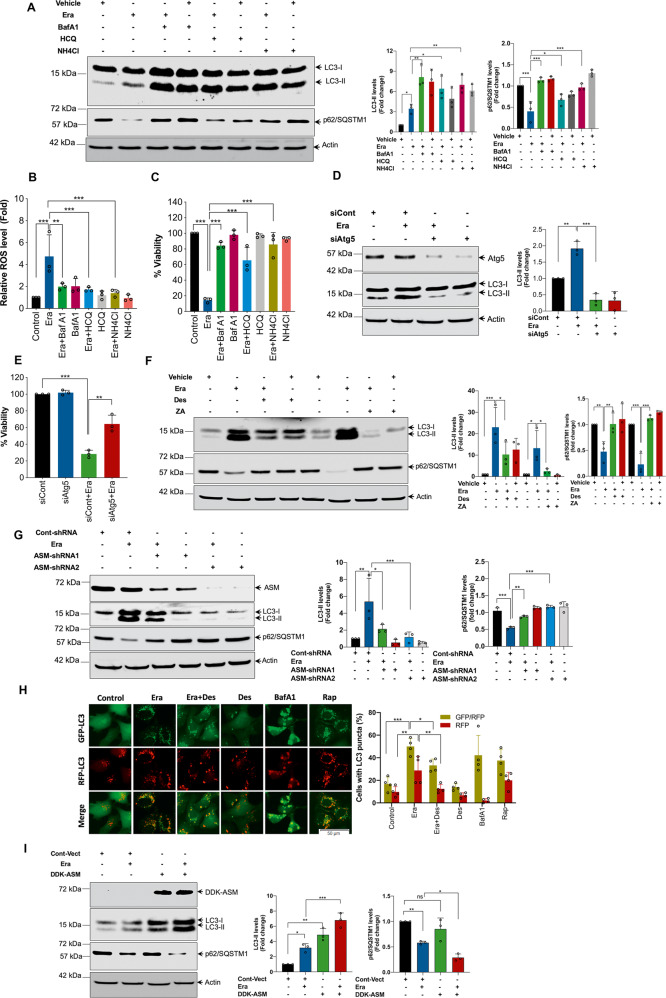
Pivotal role of ASM in the regulation of Era-induced autophagy for the execution of ferroptosis. HT-1080 cells were treated with Era (10 μM) for 12 h in the presence or absence of BafA1 (200 nM), HCQ (10 μM), and NH_4_Cl (20 mM). Following the treatment, **A** Western blot analysis of indicated proteins were carried out. Relative density of protein bands were quantified, normalized to actin of each group, and fold changes were presented in histogram from three independent experiments. Significant differences, *p < 0.05, **p < 0.01, and ***p < 0.001, **B** ROS assay by using DCFH-DA, and **C** cell viability by MTT were assessed. Significant differences, **p < 0.01 and ***p < 0.001. HT-1080 cells were transiently transfected with siAtg5 followed by exposure to Era (10 μM) for 12 h. Following the treatment, **D** Western blot analysis of indicated proteins were performed. Relative density of each protein bands were quantified, normalized to actin of each group, and fold changes were presented in histogram from three independent experiments. Significant differences, **p < 0.01, and ***p < 0.001, and **E** cell viability by MTT was assessed. Significant differences, **p < 0.01, and ***p < 0.001. HT-1080 cells were treated with Era (10 μM) in the presence or absence of **F** Des (10 μM) and ZA (20 μM) and **G** ASM-shRNA. Following the treatment, Western blot analysis of indicated proteins were analyzed. Relative density of protein bands were quantified, normalized to actin of each group, and fold changes were presented in histogram from three independent experiments. Significant differences, *p < 0.05, **p < 0.01, and ***p < 0.001. **H** HT-1080 cells expressing ptfLC3 were treated with Era (10 µM) for 12 h in the presence or absence of Des (10 μM). Fluorescent images were captured at ×20 magnification using fluorescent microscopy. Rapamycin (1 μM) was used as a positive and BafA1 (250 nM) was used as a negative control for the autolysosome formation. The number of autophagosomes represented by yellow puncta and autolysosomes represented by red puncta in merged images. Significant differences, *p < 0.05, **p < 0.01, and ***p < 0.001. **I** HT-1080 cells were stably transfected with pCMV6-DDK-ASM followed by treatment with 5 μM Era for 12 h. Following the treatment, Western blot analysis of indicated proteins were carried out. Relative density of protein bands were quantified, normalized to actin of each group, and fold changes were presented in histogram from three independent experiments. Significant differences, *p < 0.05, **p < 0.01, and ***p < 0.001.

Autophagy is a lysosomal degradative pathway that plays a complex role in various physiological and pathological conditions. Microtubule-associated protein light chain 3-II (LC3-II) conversion, p62/SQSTM1 degradation, and autolysosome formation are three well-documented hallmarks of autophagy. Several recent studies have revealed that ferroptosis is accompanied by the activation of autophagy^13,43–46^. Consistent with these reports, commonly used autophagy inhibitors, such as BafA1 (200 nM), HCQ (10 μM), and NH_4_Cl (20 mM) significantly enhanced Era-induced LC3-II accumulation (autophagic flux) and inhibited p62/SQSTM1 degradation (Fig. 5A), suggesting that at least a portion of Era-induced autophagosomes were degraded in the lysosomes. BafA1 and HCQ are potent inhibitors of late stage autophagy act by preventing autophagosomes fusion with lysosomes, whereas NH_4_Cl acts by raising the luminal pH of intracellular vesicles, preventing the activation of degradative enzymes inside lysosomes. Interestingly, all the autophagy inhibitors also significantly inhibited Era-induced ROS generation, and cell death (Fig. 5B, C). Moreover, siRNA-mediated knock-down of Atg5, inhibited the Era-induced LC3-II accumulation and cell death (Fig. 5D, E). Similar to Era-treatment, both RSL3-induced and FIN56-induced ferroptosis were also inhibited by BafA1 in Calu-1 cells (Supplementary Fig. 4), suggesting that autophagy plays a critical role in the facilitation of ferroptosis.

The functional importance of ASM in autophagosomes formation is well-established^47–49^. To check if ASM regulates ferroptosis via autophagy-dependent manner, we measured LC3-II conversion, p62/SQSTM1 degradation, and autolysosomes formation in ASM inhibited or depleted cells in the presence or absence of Era. Inhibiting ASM activity by Des and ZA or knock-down of ASM expression by means of two non-overlapping shRNAs significantly inhibited the Era-induced LC3-II conversion and p62/SQSTM1 degradation (Fig. 5F, G). Moreover, ptf-LC3-HT-1080 cells treated with Era (10 μM) exhibited both yellow (representing autophagosomes) and red (representing autolysosome) puncta formation, both of which were not detected in the control group (Fig. 5H). Likewise, abundant yellow and red puncta were seen in rapamycin-treated cells, which was used as a positive control for the autolysosome formation (Fig. 5H). In contrast, BafA1, which inhibits acidification inside the lysosome and thus impairs the autolysosome maturation, only augmented yellow puncta (Fig. 5H). Interestingly, Des significantly inhibited both yellow and red puncta formation induced by Era (Fig. 5H), thereby, suggesting that ASM-mediated autophagy plays a critical role in ferroptosis. To decipher the role of ASM further, we examined whether ASM overexpression potentiates Era-induced autophagy-mediated ferroptosis. Era (5 μM) treatment of ASM-overexpressing HT-1080 cells resulted in a marked increase in LC3-II conversion and p62/SQSTM1 degradation (Fig. 5I). Surprisingly, ASM overexpression alone was sufficient to induce autophagosomes formation as evidenced by increased LC3-II expression, hence, indicating that ASM itself might very well be playing an important role in the facilitation of autophagy-mediated ferroptosis. Together, these findings indicate that ASM-mediated autophagy is essential for the facilitation of ferroptosis.

### ASM is required for the autophagic degradation of GPX4 in ferroptosis

**Fig. 6:**
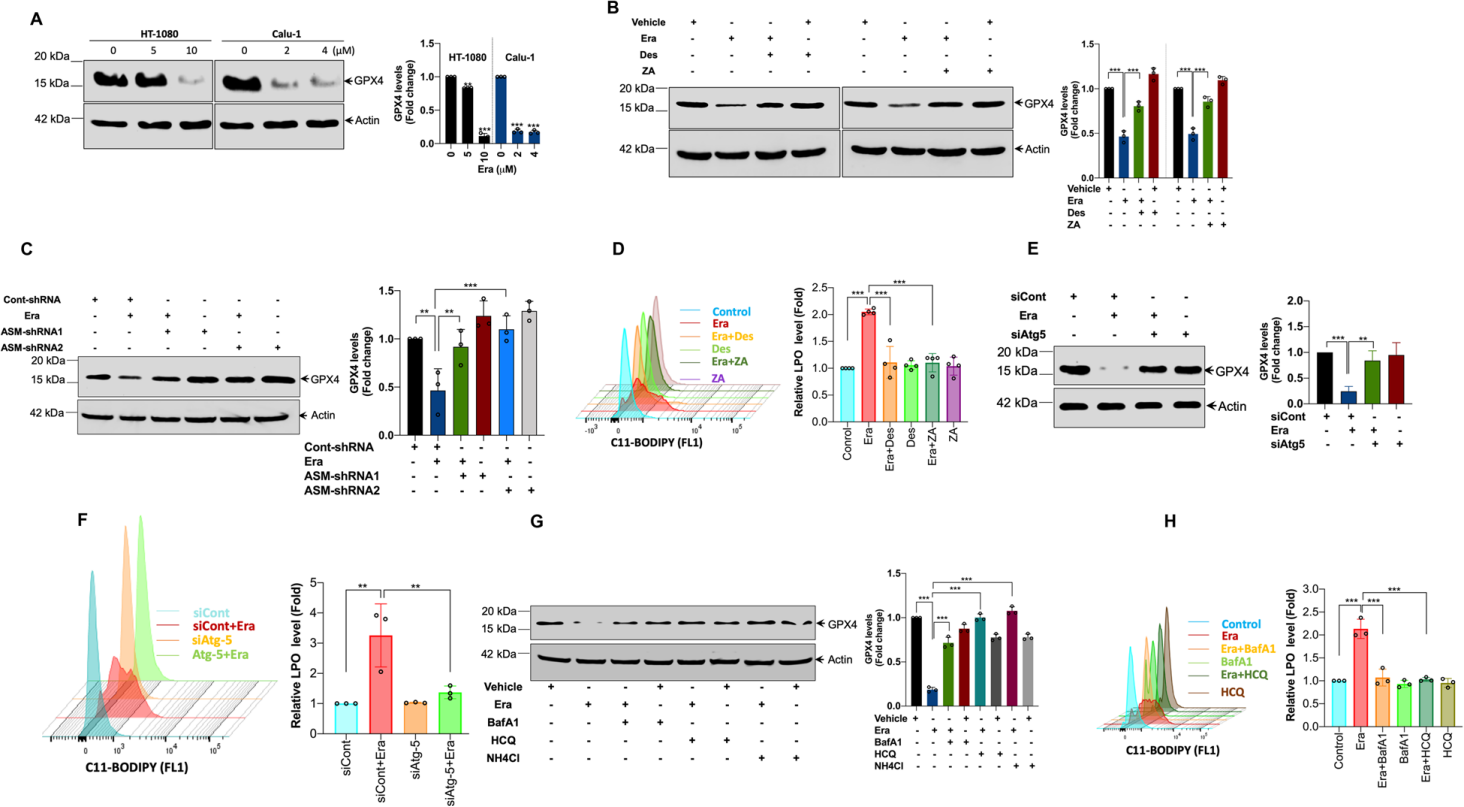
Contribution of ASM to the autophagic degradation of GPX4 for the execution of ferroptosis. **A** HT-1080 and Calu-1 cells were treated with indicated concentrations of Era for 12 h. Following the treatment, Western blot analysis was carried out. Relative density of protein bands were quantified, normalized to actin of each group, and fold changes were presented in histogram from three independent experiments. Significant differences, **p < 0.01 and ***p < 0.001. HT-1080 cells were treated with Era (10 μM) for 12 h in the presence or absence of **B** Des (10 μM) and ZA (20 μM) and **C** ASM-shRNA. Western blot analysis was carried out. Relative density of each protein bands were quantified, normalized to actin of each group, and fold changes were presented in histogram from three independent experiments. Significant differences, **p < 0.01 and ***p < 0.001. **D** HT-1080 cells were treated with Era (10 μM) for 12 h in the presence or absence of Des (10 μM) and ZA (20 μM). Following the treatment, cells were stained with BODIPY 581/591 C11 dye and LPO was detected by flow cytometry. Bar graph shows relative levels of LPO (*n* = 4). Significant differences, ***p < 0.001. HT-1080 cells were transiently transfected with siAtg5 followed by exposure to Era (10 μM) for 12 h. Following the treatment, **E** Western blot analysis was performed. Relative density of protein bands were quantified, normalized to actin of each group, and fold changes were presented in histogram from three independent experiments. Significant differences, **p < 0.01 and ***p < 0.001 and **F** cells were stained with BODIPY 581/591 C11 dye and LPO was detected by flow cytometry. Bar graph shows relative levels of LPO (*n* = 3). Significant differences, **p < 0.01. HT-1080 cells were treated with Era (10 μM) for 12 h in the presence or absence of BafA1 (200 nM), HCQ (10 μM), and NH_4_Cl (20 mM). Following the treatment, **G** Western blot analysis was carried out. Relative density of protein bands were quantified, normalized to actin of each group, and fold changes were presented in histogram from three independent experiments. Significant difference, ***p < 0.001 and **H** cells were stained with BODIPY 581/591 C11 dye and LPO was detected by flow cytometry. Bar graph shows relative levels of LPO (*n* = 3). Significant differences, ***p < 0.001.

**Fig. 7:**
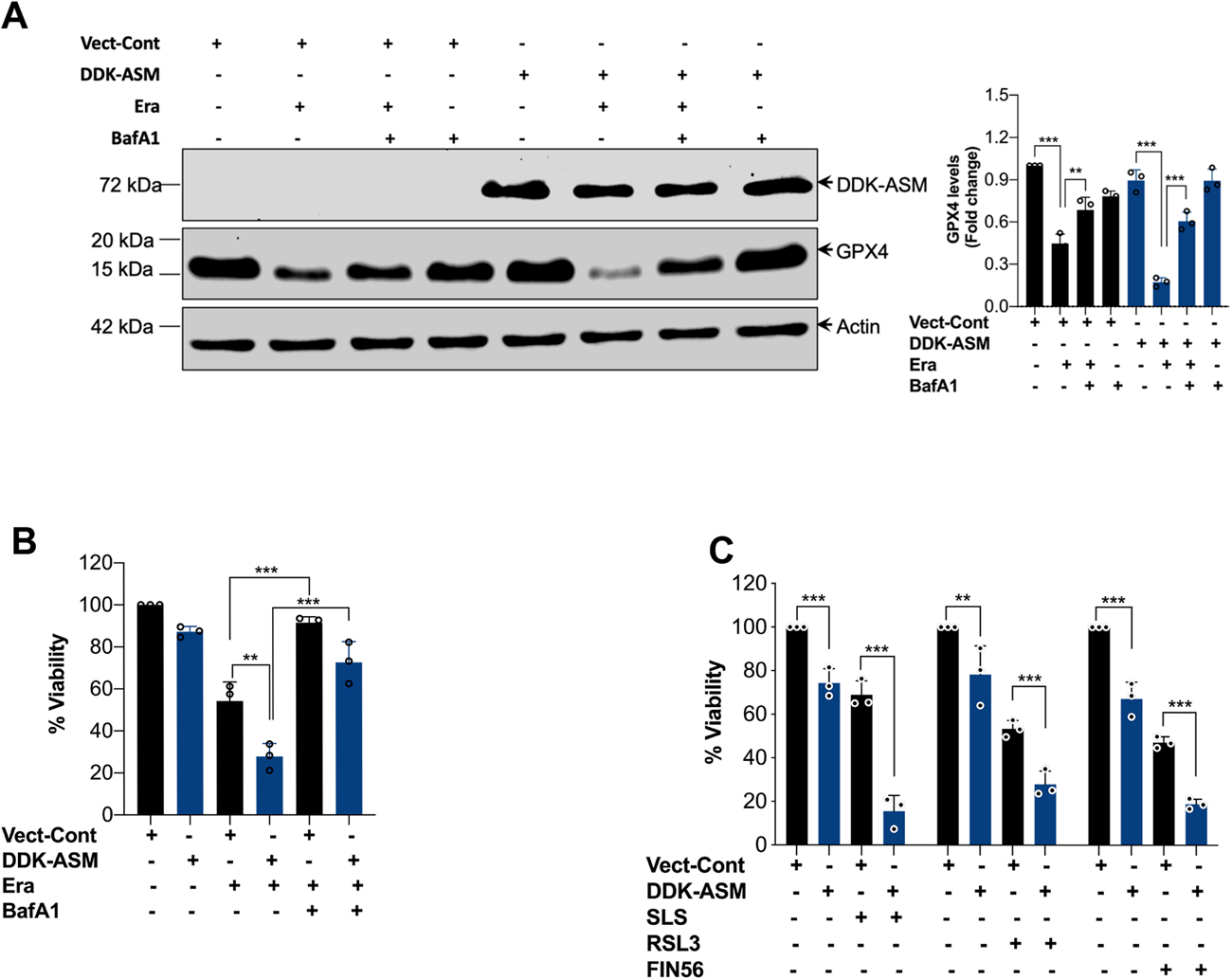
Overexpression ASM sensitizes Era-induced GPX4 degradation and FINs-induced ferroptosis. HT-1080 cells were transfected with pCMV6-DDK-ASM followed by treatment with the Era (5 μM) for 12 h in the presence or absence of BafA1 (200 nM). Following the treatment, **A** Western blot analysis of indicated proteins were carried out. Relative density of protein bands were quantified, normalized to actin of each group, and fold changes were presented in histogram from three independent experiments and **B** cell viability assessed by MTT assay. Data shown are mean ± SD (*n* = 3). Significant differences, **p < 0.01 and ***p < 0.001. **C** HT-1080 cells were transfected with pCMV6-DDK-ASM followed by treatment with SLS (0.5 mM for 24 h), RSL3 (1 μM for 12 h), and FIN56 (2 μM for 12 h). Following the treatment, cell viability was assessed by MTT assay. Data shown are mean ± SD (*n* = 3). Significant differences, **p < 0.01 and ***p < 0.001.

Degradation of GPX4 (a central defense enzyme against LPO) is crucial for LPO in many instances of ferroptosis^7–9^. Consistent with the previous reports, all tested FINs dose-dependently increased GPX4 degradation in both HT-1080 and Calu-1 cells (Fig. 6A and Supplementary Fig. 5A). To establish the direct link between ASM activation and ferroptosis, we sought to clarify whether inhibition of ASM expression or its enzymatic activity might suppress the GPX4 degradation and LPO accumulation. Surprisingly, both genetic and pharmacological inhibition of ASM significantly diminished Era-induced GPX4 degradation (Fig. 6B, C) and LPO accumulation (Fig. 6D) in HT-1080 cells. Interestingly, ASM inhibitors were also able to protect GPX4 degradation and ferroptosis induced by RSL3 and SLS in both HT-1080 and Calu-1 cells (Supplementary Figs. 5B, C and 2C, D). These results suggest that ASM inhibitors antagonize ferroptosis by preserving physiological levels of active GPX4. To further dissect how ASM regulates the degradation of GPX4, we treated cells with proteasome and autophagy inhibitors in HT-1080 cells prior to Era treatment. We found that genetic (Fig. 6E, F) or pharmacological (Fig. 6G, H) inhibition of autophagy led to noticeable protection from GPX4 degradation and LPO accumulation. By contrast, no significant protection from GPX4 degradation and cell viability was observed in cells treated with a proteasome inhibitor MG132 (Supplementary Fig. 5D, E). Next, ASM-overexpressing HT-1080 and HeLa cells were treated with Era and tested for GPX4. The results show that overexpression of ASM sensitized Era-induced GPX4 degradation and loss of viability in both cell types (Fig. 7A, B; Supplementary Fig. 6A, B). Interestingly, inhibition of autophagy protects ASM-induced sensitization of GPX4 degradation and ferroptosis (Fig. 7A, B). Furthermore, ferroptosis induced by other classes of FINs such as SLS, RSL3, and FIN56 was also significantly potentiated in the presence of ASM (Fig. 7C). Next, we checked the inhibition ROS production could protect Era-induced autophagy and GPX4 degradation. Since, inhibition of ASM reduces ROS production and vice versa, we finally examined the effect of ROS inhibitor on Era-induced autophagic GPX4 degradation. Interestingly, ROS inhibition prevented the Era-induced GPX4 degradation and autophagy (Supplementary Fig. 6C). Taken together, these findings strongly imply that ASM-mediated redox amplification regulates the autophagic degradation of GPX4, leading to ferroptosis.

## Discussion

The ASM-ceramide pathway has established cellular signaling roles in a variety of physiological functions. The pathway is also implicated in a variety of human disorders including cancer, sepsis, and neurological disorders^50^. Although, apoptosis and autophagy inducing tumoricidal functions of ASM have been significantly studied, the role and mechanism of action of ASM in the induction of ferroptosis has never been explored^30,41,42,47–49^. In this study, we report ASM as a novel and critical regulator of ferroptosis induction. Mechanistically, ASM-mediated redox amplification contributes to the autophagic degradation of GPX4, finally leading to LPO and ferroptosis.

In this study, we demonstrate that Era triggers selective activation of ASM leading to significant ceramide generation in HT-1080 cells. ASM-mediated ceramide production parallels the sensitivity of HT-1080 cells to Era, based on levels of ferroptosis markers, such as GSH depletion, ROS accumulation, and cell death. The fact that the kinetics of ASM activation, ceramide generation, and ferroptosis parallel each other suggests a critical involvement of ASM–ceramide pathway in the ferroptosis process. Ceramide increase of a similar magnitude has recently been reported in piperazine Era-induced ferroptosis in HT-1080 cells^37^. Simultaneously, ASM-mediated ceramide accumulation has also been reported in glutamate-induced ferroptosis of oligodendrocytes^33^. Our data demonstrate that specific inhibitors of ASM (Des and ZA) and knock-down ASM using shRNAs significantly attenuated Era-induced ferroptosis. It should be noted that ASM inhibitors also protected ferroptosis induced by other FINs such as SLS and RSL3. In the present study, we also demonstrate that Era-induced ASM activation is triggered by the GSH depletion and extracellular supplementation of GSH or its precursor NAC completely inhibits the Era-induced ASM activation, ceramide generation, and ferroptosis.

ROS accumulation is one of the indispensable hallmarks of ferroptosis^3,4,51^. Studies have shown that ROS can oxidize C-terminal Cys629 residue of ASM, leading to its dimerization and activation^52,53^. In support to this, genetic silencing of the NADPH oxidase subunit gp91phox was shown to inhibit the activation of ASM^54^. Meanwhile, other studies have also shown that ASM activation by death receptors, glutamate, or pathogens was inhibited by ROS scavengers such as Tiron, NAC, DPI, Cat, or SOD^33,41^. These results point to the existence of a positive cyclic correlation between ASM activation and NADPH oxidase-dependent ROS generation^40,41,55^. For instance, ASM activation causes ceramide generation, leading to the formation of ceramide-enriched membrane platforms. NADPH oxidase clusters and activates in these membrane platforms, leading to ROS generation. Once generated, ROS further activates ASM, resulting in the formation of more ceramide and the cycle repeats. In the present study, we demonstrate that Era-induces NADPH oxidase-derived ROS accumulation, which was significantly, but not completely, inhibited by ASM inhibitors. Simultaneously, antioxidants such as GSH, NAC, DPI, and Cat significantly inhibited Era-induced ASM activation. Taken together, these data point to the existence of a positive feedback loop regulation between NADPH oxidase-derived ROS accumulation and ASM activation in the facilitation of ferroptosis.

Originally, ferroptosis was described as a unique cell death process initiated and executed by novel regulators^3^. Recently, it was found that classical FINs require autophagy machinery to execute ferroptosis^38,43–45^. In particular, certain types of selective autophagy, such as nuclear receptor coactivator 4 (NCOA4)-mediated ferritinophagy, RAB7A-dependent lipophagy, and p62/SQSTM1-dependant clockophagy promote ferroptotic cell death via the upregulation of iron-mediated oxidative stress and LPO^43–46,56^. Ceramide has been shown to play a crucial role in the execution of autophagy initiated by various cytotoxic and other anticancer agents^21,57^. In addition, there is ample evidence showing that ASM-mediated ceramide formation significantly correlates with autophagy induction in a wide range of cancer cells^47,48,58,59^. Our findings indicate that Era-induced autophagy is critical for its ferroptotic activity. Both pharmacological inhibition and genetic knock-down of ASM significantly protected cells from Era-induced autophagy and ferroptosis. Meanwhile, overexpression of ASM profoundly sensitized these cells to Era-induced autophagy and ferroptosis. Collectively, these findings suggest that ASM plays a pivotal role in the induction of autophagy-mediated ferroptosis.

GPX4 is a central defense enzyme against LPO. GPX4 degradation is an indispensable signaling event in the execution of ferroptosis^4,5,7,8^. Classical FINs such as Era, SLS, RSL3, and FIN56, cause GPX4 degradation and devastating LPO, leading to ferroptosis^12–15^. The mechanism as to how these drugs induce GPX4 degradation remains unclear. We demonstrate a distinct mechanism through which ASM induces GPX4 degradation in response to ferroptosis activators. Mechanistically, we revealed that ASM is required for autophagic degradation of GPX4 by amplifying Era-induced ROS production. Inhibition of ASM resulted in the down-regulation of Era-induced ROS production and autophagy, which led to the inhibition of GPX4 degradation, and LPO and increase of cellular survival. With the increasing recognition of the fundamental role of LPO in ferroptosis, and the possible contribution of ferroptosis to many pathological contexts such as neurodegeneration and ischemia-reperfusion injury, strategies aiming at the inhibition of LPO are emerging as attractive cytoprotective strategies^4^. In this context, the identification of ASM as an important regulator of ferroptosis is of higher importance.

**Fig. 8:**
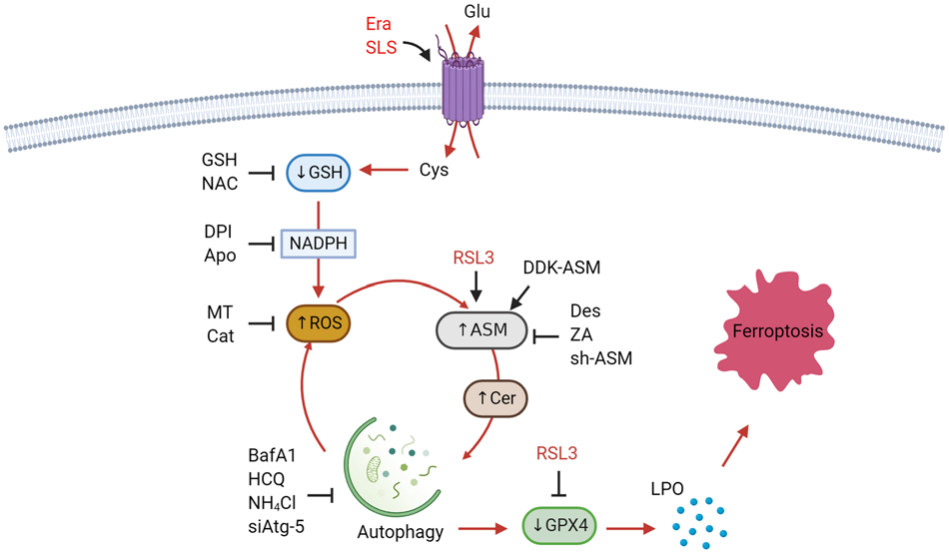
Schematic depiction of ASM-evoked execution of ferroptosis. Glu glutamate, Cys cystine, Apo apocynin, MT Mito-TEMPO, Cer ceramide. Please refer to the text for additional details.

In summary, we have identified a regulatory signaling pathway mediated by ASM that positively controls ferroptosis via activating the autophagic degradation of GPX4 (Fig. 8). This ASM-dependent ferroptosis pathway may represent a potential target for the development of pharmacological agents that enhance or inhibit ferroptosis signaling pathways.

## Supplementary information

Supplementary Figure 1

Supplementary Figure 2

Supplementary Figure 3

Supplementary Figure 4

Supplementary Figure 5

Supplementary Figure 6

Supplemental legend
