# Supplementary material for: Acid sphingomyelinase-dependent autophagic degradation of GPX4 is critical for the execution of ferroptosis
Source: Cell Death Dis. 2021 Jan 7;12(1):26. doi: 10.1038/s41419-020-03297-w (PMC7791123; doi:10.1038/s41419-020-03297-w)
Supplement: Supplementary file 7 — Supplemental legend [file 41419_2020_3297_MOESM7_ESM.docx]

**Supplementary figure legends:**

**Supplementary Figure 1. Ceramide is generated during the process of ferroptosis.** HT-1080 cells treated with RSL3 (2 μM) and FIN56 (4 μM) for 12 h. Calu-1 cells treated with Era (2 μM), RSL3 (2 μM), and FIN56 (4 μM) for 12 h. Ceramide levels were measured by using HPLC. Data shown are mean± SD (*n*=3). Significant differences, **p*<0.05, and ***p*<0.01.

**Supplementary Figure 2. ASM is involved in FINs-induced ferroptosis.** (A) Calu-1 cells were treated with Era (2 μM) for 12 h in the presence or absence of Des (10 μM) and ZA (20 μM). Following the treatment, live-dead assay was performed. (B) HeLa (20 μM for 24 h) and Calu-1 (2 μM for 12 h) cells were treated with Era in the presence or absence of Des (10 μM) and ZA (20 μM). Cell viability was measured by MTT assay. Data shown are mean± SD (*n*=3). Significant difference ****p*<0.001. HT-1080 and Calu-1 cells were treated with (C) SLS (1 mM for 24 h) and (D) RSL3 (2 μM for 12 h) in the presence or absence of Des (10 μM). Cell viability was measured by MTT assay. Data shown are mean± SD (*n*=3). Significant difference ****p*<0.001.

**Supplementary Figure 3. ROS scavengers inhibit Era-induced ferroptosis.** HT-1080 cells were treated with Era (10 μM) in the presence or absence of MT (10 μM) and Cat (2000 Units/ml). Following the treatment, (**A**) ROS assay by using DCFH-DA and (**B**) cell viability by MTT assay were performed. Data shown are mean± SD (*n*=3). Significant differences, ***p*<0.01 and ****p*<0.001.

**Supplementary Figure 4. Autophagy inhibitor inhibits FINs-induced ferroptosis.** Calu-1 cells were treated with RSL3 (4 μM) and FIN56 (2 μM) in the presence or absence of BafA1 (100 nM). Following the treatment, cell viability was assessed. Significant differences, ***p*<0.01, and ****p*<0.001.

**Supplementary Figure 5. Essential role of ASM in the autophagic degradation of GPX4 for the execution of ferroptosis (A)** HT-1080 and Calu-1 cells were treated with indicated concentrations of SLS (24 h), RSL3 (12 h), and FIN56 (12 h). Following the treatment, Western blot analysis was carried out. Relative density of protein bands were quantified, normalized to actin of each group, and fold changes were presented in histogram from three independent experiments. Significant differences, ****p*<0.001. HT-1080 and Calu-1 cells were treated with (**B**) RSL3 (2 μM) and (**C**) SLS (1 mM) for 12 h in the presence or absence of Des (10 μM). Following the treatment, Western blot analysis was carried out. Relative density of protein bands were quantified, normalized to actin of each group, and fold changes were presented in histogram from three independent experiments. Significant differences, **p*<0.05, ***p*<0.01, and ****p*<0.001. HT-1080 cells were treated with Era (10 μM) or RSL3 (4 μM) for 12 h in the presence or absence of MG132. Following the treatment, (**D**) Western blot analysis was carried out. Actin was used as a loading control. Blots shown are representative of three independent experiments and (**E**) cell viability was assayed by MTT assay. Data shown are mean± SD (*n*=3). Significant differences, ****p*<0.001.

**Supplementary Figure 6. ASM sensitizes Era-induced GPX4 degradation and ferroptosis.** HeLa cells were transiently transfected with pCMV6-DDK-ASM followed by treatment with the indicated concentration of Era for 24 h. Following the treatment, (**A**) Western blot analysis of indicated proteins were carried out. Relative density of protein bands were quantified, normalized to actin of each group, and fold changes were presented in histogram from three independent experiments and (**B**) cell viability assessed by MTT assay. Data shown are mean± SD (*n*=3). Significant differences, ****p*<0.001. (**C**) HT-1080 cells were treated with erastin for 12 h in the presence or absence of NAC (5 mM). Following the treatment, Western blot analysis of the indicated proteins were carried out.
